# Visual search in depth: The neural correlates of segmenting a display into relevant and irrelevant three-dimensional regions

**DOI:** 10.1016/j.neuroimage.2015.07.052

**Published:** 2015-11-15

**Authors:** Katherine L. Roberts, Harriet A. Allen, Kevin Dent, Glyn W. Humphreys

**Affiliations:** a Department of Psychology, University of Warwick, Coventry, UK; b School of Psychology, University of Nottingham, Nottingham, UK; c Department of Psychology, University of Essex, Colchester, UK; d Department of Experimental Psychology, University of Oxford, Oxford, UK

**Keywords:** Visual search, Segmentation, Depth, fMRI

## Abstract

Visual perception is facilitated by the ability to selectively attend to relevant parts of the world and to ignore irrelevant regions or features. In visual search tasks, viewers are able to segment displays into relevant and irrelevant items based on a number of factors including the colour, motion, and temporal onset of the target and distractors. Understanding the process by which viewers prioritise relevant parts of a display can provide insights into the effect of top-down control on visual perception. Here, we investigate the behavioural and neural correlates of segmenting a display according to the expected three-dimensional (3D) location of a target. We ask whether this segmentation is based on low-level visual features (e.g. common depth or common surface) or on higher-order representations of 3D regions. Similar response-time benefits and neural activity were obtained when items fell on common surfaces or within depth-defined volumes, and when displays were vertical (such that items shared a common depth/disparity) or were tilted in depth. These similarities indicate that segmenting items according to their 3D location is based on attending to a 3D region, rather than a specific depth or surface. Segmenting the items in depth was mainly associated with increased activation in depth-sensitive parietal regions rather than in depth-sensitive visual regions. We conclude that segmenting items in depth is primarily achieved via higher-order, cue invariant representations rather than through filtering in lower-level perceptual regions.

## Introduction

Being able to selectively attend to relevant aspects of the world is critical for efficient information processing (Broadbent, 1958, Tsotsos, 1990). Prioritisation of items of interest can be based on low-level visual features such as colour (Wolfe et al., 1989) or motion (McLeod et al., 1988), or on more complex features such as common temporal onset (Watson and Humphreys, 1997). Understanding the process by which prioritisation is achieved can provide insights into the mechanisms by which cognitive control influences perceptual representations. Neuroimaging has revealed that a region of the precuneus is involved in segmenting a scene into relevant and irrelevant items based on different features (motion, temporal onset; Dent et al., 2011). Activation is also found in the relevant feature-specific regions, such as those representing motion (Dent et al., 2011). Here, we extend this work to investigate the mechanisms involved in selectively attending to items in a relevant 3D region of space. Segmenting a scene into relevant and irrelevant 3D regions can help distinguish steps, kerbs and other hazards, or help find objects in a crowded shop display. We ask whether the same precuneus region involved in segmenting items by motion and time is also involved in segmenting items in depth. We also ask if segmenting items in depth is associated with activation in visual areas tuned to disparity or surfaces, or parietal regions containing higher-order 3D representations.

Visual search tasks have proved to be a valuable tool for evaluating the ability to segment a visual scene into relevant and irrelevant regions. In visual search tasks, participants search for a target item while ignoring irrelevant (distractor) items. When the target is defined by a single feature (e.g. colour), search is highly efficient and not dependent on the number of distractors in the display (‘pop-out’ search). When the target is defined by a conjunction of features (e.g. colour and form), search time increases with increasing numbers of non-target distractors (Treisman and Gelade, 1980). These data indicate that search is facilitated if participants can segment the scene into relevant and irrelevant items, and can direct their attention to only the relevant subset of items (see Wolfe and Horowitz, 2004, for a review). When participants are able to segment the search display in this way, search is more efficient, with search time reflecting the number of distractors in the attended subset rather than the number of distractors in the entire display. This has been demonstrated with temporal segmentation (‘preview search’, in which a subset of distractors are presented in advance; Theeuwes, Kramer, & Atchley, 1998; Watson and Humphreys, 1997), colour segmentation (Wolfe et al., 1989), and motion segmentation (in which a subset of items are moving, Dent et al., 2011, McLeod et al., 1988; von Muhlenen & Muller, 2000). There is also evidence that depth cues can be used to segment items in a display (Finlayson et al., 2013, Nakayama and Silverman, 1986), with participants able to perform an efficient ‘pop-out’ search within an attended depth plane.

Numerous studies have demonstrated that attention can be directed to a specific location in 3D space (e.g. Anderson and Kramer, 1993, Nakayama and Silverman, 1986). However, there is debate over whether attention can be directed to a specific depth (disparity), or whether attention is in fact allocated to surfaces within 3D space (He and Nakayama, 1995). It also seems that there must be considerable separation between the depth planes in order for them to be separately attended (more than 6 min of arc), even though perceptual stereo thresholds are considerably smaller (on the order of seconds rather than minutes; de la Rosa et al., 2008). He and Nakayama (1995) found that participants were unable to attend to items that shared a common disparity if the individual items were tilted forwards or backwards, preventing them from appearing to fall on a common surface. In contrast, the participants were able to attend to items on a plane that was tilted in depth, so that the items formed a surface but were at different disparities. It may be that separate mechanisms are engaged when attention is directed to a specific depth or to a surface in depth. He and Nakayama (1995) found that increasing the separation in depth (disparity) between target and distractor items impaired selective attention when the items were on different planes, but had no effect when those same items appeared to be on a surface that was tilted in depth.

Wheatley et al. (2004) suggested that different surfaces are preattentively segregated. Participants were asked to detect the number of targets that differed in depth from background items. Search was efficient when the target items fell on the same surface, even when it was tilted in depth, but inefficient when items appeared on different depth planes. Preattentive segregation of depth planes has also been demonstrated in multiple-object tracking tasks (Haladjian et al., 2008). Viswanathan and Mingolla (2002), for example, found that performance on a tracking task was improved when targets and distractors were presented in two depth planes rather than one, and when items appeared on tilted surfaces. Interestingly, unlike in the visual search studies described above, a benefit was also found when items appeared within depth-defined volumes. This finding is consistent with results on flanker interference (Anderson and Kramer, 1993), where there is an attentional gradient in depth, with flanker interference decreasing as the separation in depth of targets and flankers increases. These studies indicate that it may be possible to selectively attend to items within a depth-defined region of space, even when those items do not form a common surface. This is in keeping with real-world examples of segmentation search, such as searching for a friend arriving at a train station where we may exclude from search (a) people who have been on the station for some time (segmentation by time/preview search); (b) people who are stationary (segmentation by motion); and (c) people who are nearer or further down the platform, who are unlikely to be coplanar (segmentation in depth).

**Fig. 1:**
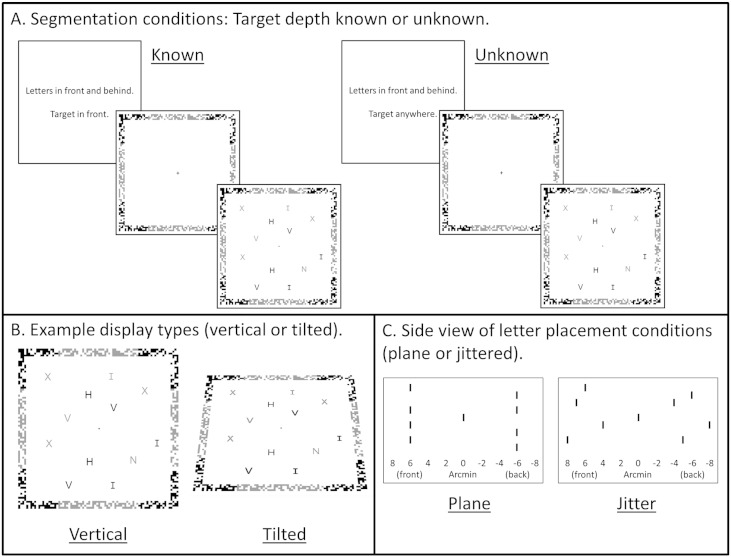
Experimental design. The task was to indicate if the display contained an N or a Z. Displays contained 8 or 16 letters. Light and dark grey indicate different depths (in front and behind fixation). Panel A. Example of the first three displays in each block. An initial screen instructs participants where stimuli will appear in that block (‘in front and behind’ or ‘on top and below’) and where the target will appear (‘target in front’, ‘target behind’, ‘target on top’, ‘target below’, ‘target anywhere’). Each trial then comprises a fixation screen and letter display. Panel B. Example vertical and tilted displays. Panel C. Side view to illustrate the plane and jitter letter placement conditions. The small lines indicate the placement in depth of letters and the fixation cross.

In the present study, we examined the neural basis of segmenting items in depth, using fMRI while participants performed a difficult search task, in which they did or did not know the likely 3D location of the target. Participants searched for a target among distractors, with items appearing in front and behind the fixation plane. Displays were identical in the two top-down segmentation conditions, the only difference being that when the 3D location of the target was known, participants could use this information to segment the scene into relevant and irrelevant items, searching only the relevant items (Factor 1. Target depth known versus unknown; Fig. 1A). Two further factors were included to separate effects of attending to depths and surfaces. Displays were either vertical (fronto-parallel) or tilted backwards 45° (Factor 2. Display type: vertical or tilted; Fig. 1B). Within the display condition, letters in front and behind fixation were either presented with a common disparity (so that letters formed planes at different depths) or within depth-defined volumes (so that letters were jittered in depth and did not form planes) (Factor 3. Letter placement: planes or jittered; Fig. 1C). Depth regions were therefore defined by either common disparity (vertical displays, planes), common surfaces (vertical and tilted displays, planes) or a depth-defined region of space only (vertical and tilted displays, jittered). Comparing activation when target depth was known versus unknown, for the different display types and letter placement conditions, allows us to isolate activation associated with selectively attending to a specific region of 3D space, whether defined by a common disparity, common surface, or depth-defined regions.

Previous work has indicated that segmentation by time and motion activates a common region of the precuneus, as well as task-specific regions (Dent et al., 2011). The precuneus is likely to be involved in maintaining a spatial representation of distractor locations, and activity in this region is correlated with the amount of benefit obtained from segmenting the display (Dent et al., 2011). During segmentation by motion, activation was also found in motion-processing areas (Dent et al., 2011). In this case, segmentation may be at least partially based on a motion filter in the feature-specific region MT/MT +, which is used to guide attention to moving items and to filter out stationary items (Ellison et al., 2007, McLeod et al., 1988). We hypothesise that segmenting items according to their 3D location will recruit the same region of the precuneus as that identified by the previous segmentation tasks, demonstrating the supramodal nature of visual segmentation in this brain region (Dent et al., 2011). We are also interested in whether segmenting items according to their 3D location leads to increased activation in visual regions sensitive to depth perception (kinetic occipital area (KO), motion area MT/MT +, and lateral occipital cortex (LO)), or in higher-order depth-sensitive regions along the intraparietal sulcus (IPS) (Preston et al., 2008). If segmenting items in depth is achieved through filtering in depth-sensitive visual regions, we might expect increased activation in the target-known condition, as is the case in MT/MT + when participants attend to motion (e.g. Dent et al., 2011, Saenz et al., 2003). An alternative possibility is that visual regions may show reduced activation due to attention being focused on only part of the display.

We are also interested in whether activity in the visual and parietal depth-sensitive regions is cue invariant, or if it depends on the cues available to target depth (i.e., common disparity, common surface or depth-defined regions). Note that we use the term ‘segmentation’ to refer to dividing a search display into relevant and irrelevant items according to a specified feature; in this case, their 3D location. This is distinct from the perceptual process of segmenting a visual scene into surfaces and objects. Similarly, ‘cues to target depth’, refers to the cues available to the participant to aid them in dividing the scene into relevant and irrelevant items (e.g. the possible range of target disparities). This is not the same as ‘cues’ available for depth perception, such as occlusion and motion parallax.

## Materials and methods

### Participants

Seventeen participants took part. Data from one participant had to be excluded due to excessive movement during the imaging session. The analyses are based on data from the remaining 16 participants (5 male, mean age 23 years (19 to 33 years), all reported being right-handed). All participants gave written informed consent and received £20 compensation.

### Stimuli and design

**Supplementary Fig. 1:**
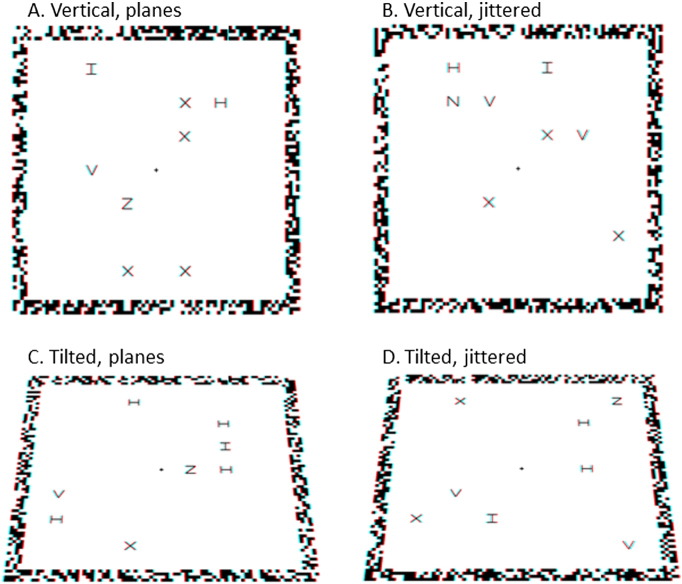

The task was to search for a target letter (Z or N) among distractor letters (H, I, V, X), and indicate with a button-press response whether the target was a Z or an N. Participants responded using the index finger of their right hand for Z, and the middle finger of their right hand to for N. The search display contained either 8 or 16 letters, with equal numbers to the left and right of the display. Stimuli were presented stereoscopically as anaglyphs using PsychToolBox 3 (Brainard, 1997) in MATLAB (The Mathworks: Natick, MA), and viewed through a pair of red/cyan glasses. Half of the displays were vertical (fronto-parallel), while the other half were tilted backwards 45° (Fig. 1B). In the vertical displays, a fixation cross was at the centre of the fixation plane and target and distractor letters presented either 6 arcmin in front and behind fixation (co-planar) or within depth-defined volumes located between 4 and 8 arcmin in front and behind fixation (jittered) (Fig. 1C). Half the letters appeared in front of the fixation plane and half appeared behind the fixation plane. Each letter measured 10 mm^2^ (visual angle = 0.88°) and was arranged on a virtual grid of 7 columns by 7 rows, with the central column empty. A frame around the letter display provided a reference to +/- 6 arcmin disparity; with either the corners or edges of the frame in front and the edges or corners behind (counterbalanced across participants). The frame measured 25.5 cm^2^ (visual angle = 22.20°). Stimuli were projected onto a screen at the back of the scanner and were viewed from a distance of approximately 65 cm via a mirror placed on the head coil. Tilted displays were the same as vertical displays, but tilted backwards 45° using OpenGL to create displays that were slanted in depth. See Fig. 1 for example displays, in which light/dark grey is used to indicate the different depths. Anaglyphs are provided in Supplementary Fig. 1 and can be viewed through red/cyan glasses.

### Procedure

Prior to the imaging session, participants were screened for adequate depth perception and practiced the task. To evaluate depth perception, participants viewed the experimental displays and indicated if the target was in front or behind fixation. Two participants were unable to reliably determine if the target letter was in front or behind fixation in the fronto-parallel displays and were excluded from the study. The remaining 17 participants correctly identified the target depth (front or back) on at least 7 out of 10 trials at each of four disparities (8, 6, 4, and 2 arcmin).

During the imaging session, participants completed three runs of the experimental task. Each run comprised 16 blocks of 10 trials, with each block preceded by a 3-s instruction window that informed participants where the letters would appear (‘letters in front and behind’ or ‘letters on top and below’) and where the target would appear (‘target in front’, ‘target behind’, ‘target on top’, ‘target below’ or ‘target anywhere’) (Fig. 1A). In each run, there were two blocks for each condition (vertical/tilted displays × plane/jittered letter placement). In the target-known conditions, targets were in front or on top in one block and behind or below in the other block. In the target-unknown conditions, targets were randomly in front or behind in both blocks. Trials randomly contained either 8 or 16 letters. On each trial, participants viewed a 1000-ms fixation cross (plus the reference frame) followed by the search display and reference frame for 2000 ms. If a response was not made within the 2-s display time, the trial was marked as incorrect. Each block therefore lasted 33 s. After every four blocks, there was a 15-s baseline block in which only a fixation cross was visible. Each run lasted 588 s in total.

Following the experimental task, we acquired a high-resolution anatomical scan and participants completed four functional localiser tasks, to identify the lateral occipital cortex (LO), kinetic occipital area (KO), human motion complex (MT/MT +), and depth-sensitive regions along the intraparietal sulcus (IPS), namely, the ventral IPS (VIPS), parieto-occipital IPS (POIPS) and dorsal IPS (DIPS). The object-processing region LO was identified by comparing the blood oxygen-level dependent (BOLD) response to intact objects relative to scrambled objects (Kourtzi and Kanwisher, 2001, Kourtzi et al., 2005). For this task, participants performed a 1-back task (press a button when an image is repeated). KO was identified as the region showing a significantly greater response to kinetic boundaries than transparent motion of a field of black and white dots (Dupont et al., 1997; cf. Larsson and Heeger, 2006). The motion-processing region MT/MT + was identified as the region showing an increased response to a coherently moving array of dots than to a static array of dots (Zeki et al., 1991). A further localiser was included to identify areas along the IPS that showed an increased response to three-dimensional shape defined by disparity and structure-from-motion cues (Orban et al., 1999, Chandrasekaran et al., 2007), but activation from this task proved too unreliable to create function ROIs for individual participants. For the KO, MT/MT + and IPS localiser tasks, participants made a button-press response to indicate when the fixation dot or cross changed colour.^1^

### MRI data acquisition

Imaging data were acquired using a Phillips 3T Achieva scanner at Birmingham University Imaging Centre. A T1-weighted 1 × 1 × 1 mm anatomical image was acquired for each participant. T2*-weighted functional echoplanar imaging data were obtained using an eight-channel SENSE head coil with a sense factor of 2. For the experimental task, data were acquired for 54 slices (2.5 mm^3^ resolution, TR = 3 s, TE = 35 ms, flip angle = 85°). For the localiser tasks, data were acquired for 28 slices with 1.5 × 1.5 × 2 mm resolution, TR = 2 s. Slices were aligned coronally and covered the occipital cortex (LO, KO, MT/MT +) or parietal lobe (IPS).

### Data analysis

Imaging data were analysed using SPM8 (Wellcome Department of Imaging Neuroscience, London; www.fil.ion.ucl.ac.uk/spm). Data were spatially realigned and unwarped to correct for motion artefact and distortions in the magnetic field, then transformed into MNI space and spatially smoothed using a Gaussian kernel of 8 mm full-width-at-half-maximum.

### Random effects and region of interest (ROI) analyses

Data were analysed using a block design. Data were modelled at the individual level with regressors for each condition (vertical-plane known, vertical-plane unknown, vertical-jitter known, vertical-jitter unknown, tilted-plane known, tilted-plane unknown, tilted-jitter known, tilted-jitter unknown) convolved with the canonical haemodynamic response function (HRF). Additional regressors were included to account for movement artifacts and the different runs of the task. A 1/512 Hz high-pass filter was applied to remove low-frequency noise. Data for each experimental condition, for each participant, were then entered into second-level whole brain and ROI analyses. The ROI analysis was conducted using MarsBaR (Brett et al., 2002). We created 5-mm spheres centred on the peak coordinates identified by the ROI localiser tasks. This was successful for the KO, LO, and MT/MT + localiser tasks. Where data were missing, due to technical difficulties or scanning-time limitations, the peak from a second-level group analysis was used instead. This was necessary for one participant for LO and four participants for MT/MT +. The peak coordinates for the group analysis were: left KO: − 33, − 80, 10; right KO: 42, − 78, 13; left LO: − 46, − 74, − 5; right LO: 48, − 72, − 5; left MT/MT +: − 44, − 74, 2; right MT/MT +: 56, − 70, − 4. Unfortunately, the IPS localiser task did not produce robust regions of activation within individuals, and so we instead used the coordinates provided in Georgieva et al. (2009) for anterior DIPS (DIPSA: − 36, − 52, 64; 36, − 50, 56), medial DIPS (DIPSM: − 22, − 64, 60; 26, − 60, 60), putative human anterior intraparietal area (phAIP: − 42, − 42, 50; 42, − 42, 52), parieto-occipital IPS (POIPS: − 20, − 78, 48; 28, − 82, 44) and ventral IPS (VIPS/V7: − 28, − 78, 34; 28, − 80, 36). For each of the spherical ROIs, we extracted the mean contrast value across the ROI for each individual participant, averaged across the left and right hemispheres and then entered these values into analyses of variance (ANOVAs). We created one additional ROI in the precuneus, centred on the coordinates from Dent et al. (2011) (10, − 56, 30).

## Results

### Behavioural data

**Fig. 2:**
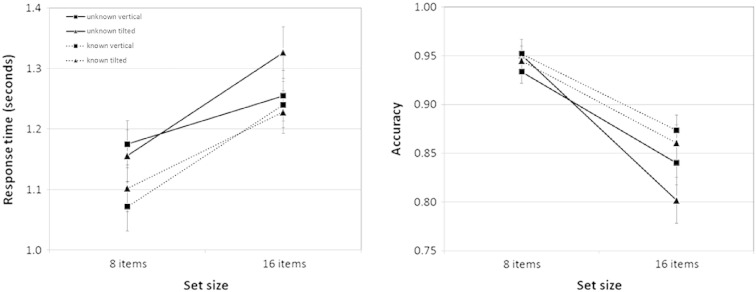
Response times and accuracy when target location was known (dotted lines) and unknown (solid lines), for vertical (squares) and tilted (triangles) displays. Error bars show standard errors.

Response times and accuracy were entered into repeated-measures analyses of variance (ANOVAs) contrasting display type (vertical or tilted), letter placement (plane or jitter), knowledge of target location (known or unknown) and set size (8 or 16 letters). If a response was not made within the 2-s display time, the trial was marked as incorrect. This occurred on 6.6% of trials. Accuracy approached ceiling and so the data were arcsine transformed prior to analysis. Median response times (RTs; correct trials only) tended to be faster and more accurate with the smaller set size (RTs: *F*[1,15] = 110.9, *p* < 0.001; accuracy: *F*[1,15] = 141.0, *p* < 0.001) and when the target location was known (RTs: *F*[1,15] = 14.2, *p* = 0.002; accuracy: *F*[1,15] = 4.2, *p* = 0.06). Although search was faster and more accurate in the target-known condition, there was no two-way interaction between set size and target knowledge that would indicate improved search efficiency (RTs: *F*[1,15] = 2.8, *p* = 0.11; accuracy: *F*[1,15] = 0.88, *p* = 0.36). There was a three-way interaction between display type (vertical or tilted), set size and target-location knowledge (RTs: *F*[1,15] = 9.1, *p* = 0.009; accuracy: *F*[1,15] = 3.6, *p* = 0.076). This was due to unusually good performance with larger set sizes in vertical displays when target location was unknown (see Fig. 2). There were no effects of whether the letters formed a plane or were jittered within depth-defined volumes. See Fig. 2 for RT and (raw) accuracy data, collapsed across the letter placement conditions.

### Neuroimaging data

#### Region of interest analysis

**Fig. 3:**
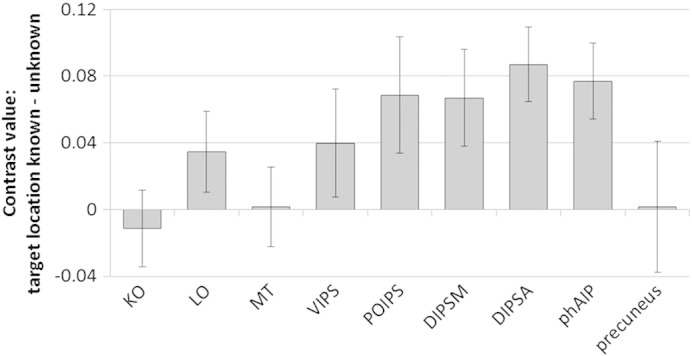
Change in activation in specific regions of interest when the location of the target was known versus unknown. Error bars show standard errors. KO, kinetic occipital; LO, lateral occipital; MT, MT/MT +; VIPS, ventral intraparietal sulcus; POIPS, parieto-occipital intraparietal sulcus; DIPSM, medial dorsal intraparietal sulcus; DIPSA, anterior dorsal intraparietal sulcus; phAIP, putative human anterior intraparietal area.

We conducted region of interest (ROI) analyses to determine whether specific cortical regions known to be sensitive to depth information were activated by segmenting items in depth. We extracted mean activation within 5-mm spherical ROIs located within left and right KO, LO, MT/MT +, VIPS/V7, POIPS, DIPSM, DIPSA, and phAIP, plus the precuneus (following Dent et al. (2011)). We then conducted ANOVAs contrasting display type (vertical, tilted), letter placement (plane, jittered) and knowledge of target location (target location known, unknown) for each ROI, averaged across left and right, using a Bonferroni correction for multiple comparisons (critical *p* = 0.0056). The results of the main effects analyses can be seen in Table 1. A number of ROIs showed increased activation when the target location was known versus unknown (Fig. 3). There were no significant interactions involving any of the factors. None of the higher-order visual areas (KO, LO, MT/MT + and VIPS/V7) showed a significant response to segmenting the items in depth (known vs unknown). There was a bigger response to knowledge of target location in parietal areas (DIPSA, DIPSM, POIPS and phAIP), with all regions showing increased activation when target location was known. This increase was statistically significant in DIPSA (*p* = 0.001) and phAIP (*p* = 0.004), but not in DIPSM (*p* = 0.036) or POIPS (*p* = 0.068). Knowledge of target location did not influence activity in the precuneus ROI. None of the ROIs showed a significant response to display type (vertical or tilted) or letter placement (planes or jittered), and there were no significant interactions between any of the factors.

#### Links between ROI activation and RT benefits from knowing target location

There were no significant correlations between the reaction-time benefit from knowing target location and the corresponding increase in activation in any of the ROIs.

**Fig. 4:**
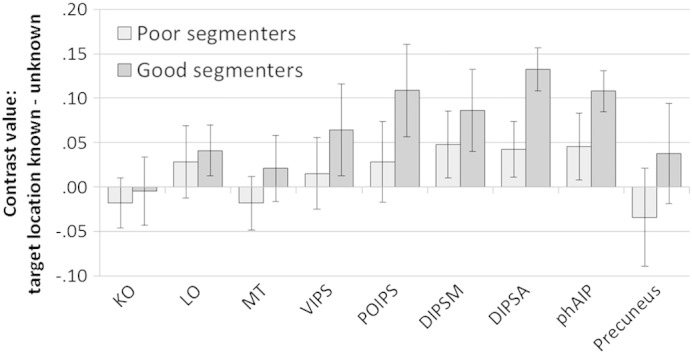
Change in activation when target location was known versus unknown, in each of the regions of interest (ROIs), for good and bad segmenters (based on a median split of reaction-time benefit). Error bars show standard errors. KO, kinetic occipital; LO, lateral occipital; MT, MT/MT +; VIPS, ventral intraparietal sulcus; POIPS, parieto-occipital intraparietal sulcus; DIPSM, medial dorsal intraparietal sulcus; DIPSA, anterior dorsal intraparietal sulcus; phAIP, putative human anterior intraparietal area.

We divided the group into good and bad segmenters based on a median split of the RT benefit when target location was known versus unknown. The results are shown in Fig. 4. Good segmenters had a greater increase in activation in all ROIs when target depth was known compared with unknown. This only reached (uncorrected) significance in DIPSA (*t*[14] = 2.3, *p* = 0.038), reflecting high variability across the small number of participants (see standard error bars in Fig. 4).

#### Whole brain analysis

**Fig. 5:**
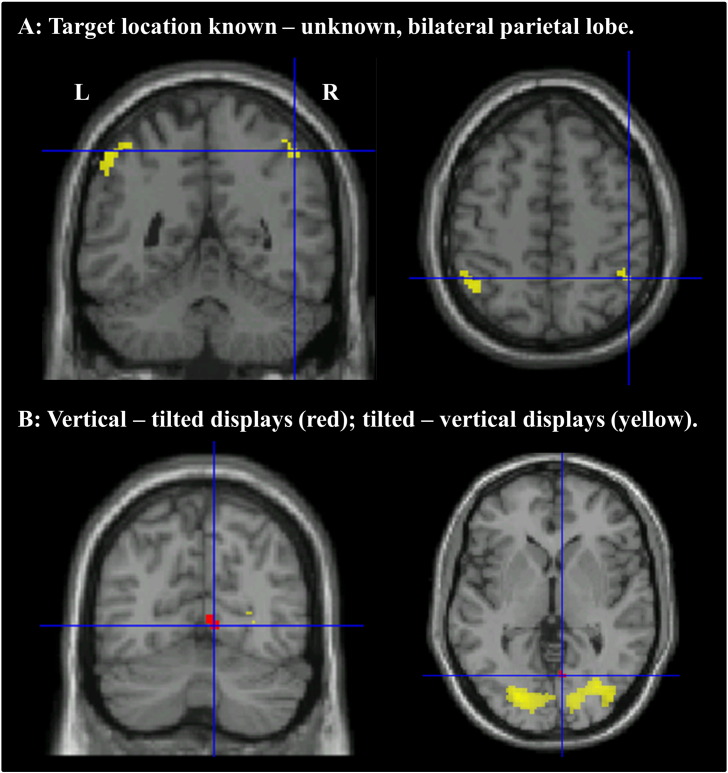
A. Parietal activation associated with knowing target location, crosshairs at 47, − 50, 52. B. Activation associated with viewing vertical versus tilted displays, crosshairs at 8, − 67, 0. *p* < 0.001 uncorrected, extent > 10 suprathreshold voxels.

A whole brain analysis was conducted to ensure that the ROI analysis had not missed any key areas of interest. No regions survived correction for multiple comparisons (FWE *p* < 0.05), but for descriptive purposes only, uncorrected results (*p* < 0.001 uncorrected, extent > 10 voxels) are shown in Table 2. We were primarily interested in regions that showed increased activation when target location was known versus unknown. This analysis revealed activation in the bilateral inferior parietal lobe centred around coordinates − 43, − 54, 55 and 47, − 50, 52 (Fig. 5). These regions were close to the ROIs in DIPSA (− 36, − 52, 64; 36, − 50, 56) and phAIP (− 42, − 42, 50; 42, − 42, 52). In addition, regions of the posterior cingulate cortex and right cerebellum showed increased activation in the location-known condition (see Table 1).

A small region of visual cortex was more engaged by vertical displays than tilted displays. This might be expected as the tilted displays subtended a smaller visual angle than the vertical displays (Fig. 1). A larger, more posterior region was more engaged by tilted displays than vertical displays (Fig. 5), perhaps due to the increased difficulty of processing the tilted letters. No regions were more engaged when the letters were in planes rather than jittered, but small regions of bilateral supramarginal gyrus were more engaged when the letters were jittered than when they were in planes.

We then looked within regions that showed increased activation when target location was known (*p* < 0.05 uncorrected) to see if that increase in activation was influenced by display type (vertical, tilted) or letter placement (plane, jittered). The right supramarginal gyrus (37 voxels, peak at 60, − 44, 38; peak *z* score = 4.06) showed a stronger response to knowing target location when the displays were vertical than when they were tilted. No regions showed a similar increase for tilted displays compared with vertical. A region of the precuneus (12 voxels, peak at 7, − 72, 38, peak *z* score = 3.61) showed increased location-known activation when the items were in planes rather than jittered, as did the cerebellum (16 voxels, peak at 14, − 74, − 38, peak *z* score = 3.77). There were no regions showing a similar response to jittered letters compared with letters in planes.

## Discussion

Participants responded more quickly and accurately when the 3D location of the target was known compared with when they viewed identical displays but the target location was unknown. This indicates that they were able to benefit from attending to the relevant 3D region.

Results of the region of interest (ROI) analyses indicate that segmenting items according to their 3D location is associated with an increase in activation in depth-sensitive regions along the intraparietal sulcus (POIPS, DIPSM, DIPSA, phAIP; Georgieva et al., 2009), particularly in the left hemisphere. These regions were more active in ‘good’ segmenters who showed a larger RT benefit from knowing target depth. Of the higher-order visual areas that are sensitive to depth (KO, LO, MT/MT +, VIPS/V7; Preston et al., 2008), none were modulated by attention to a 3D region. This pattern of activation, where knowledge of target depth affects activity in depth-sensitive parietal regions but not in depth-sensitive visual regions, suggests that segmenting items in depth is achieved through attentional selectivity in higher-order areas rather than filtering in perceptual regions.

Although the stimuli themselves were identical on target-known and target-unknown trials, there may have been differences in participants’ search behaviour. When target location was known, participants may have been able to search a smaller region of space with fewer vergence eye movements, smaller shifts of attention, and faster RTs. Note that all of these factors would have led to a decrease in activation in fronto-parietal regions responsible for attention and eye movements, not an increase (Alkan et al., 2011, Corbetta and Shulman, 2002). It is therefore striking that we found increased activation in parietal regions despite reduced demands on the fronto-parietal network. In addition, when the stimuli were tilted in depth, the attended stimuli spanned different disparities in both the known and unknown conditions, minimising any difference in the amount of space searched. Despite this, we found no interaction between display type (vertical, tilted) and knowledge of target location in any of the ROIs.

Our results suggest that segmenting items in depth is dependent on the 3D location of the attended items rather than a specific disparity or surface. The behavioural and neuroimaging analyses showed very similar results for segmenting items in vertical and tilted displays, and for segmenting items that formed planes or were jittered in depth. Segmenting the items was therefore not achieved by attending to a specific disparity, which would only be beneficial for vertical displays with letters in planes, or by attending to surfaces, which would only be beneficial when letters were in planes. It may have been the case that there were insufficient items at each depth for them to form a convincing percept of a surface. Participants may have gained a stronger benefit from knowing the target depth if surfaces had been more clearly defined. However, it is clear that participants were able to attend to items in a particular 3D region of space, without those items needing to form a plane or surface. This is in keeping with real world examples where knowing the likely 3D location of a target is beneficial, for example, when searching for people, cars, luggage or books, which would not normally form planes or surfaces.

Recent behavioural research (Finlayson et al., 2013) showed that knowing target depth did benefit visual search, but the authors concluded that there was no evidence for preattentive segmentation of the display in the way that has been found for other visual features, such as colour or motion (Anderson et al., 2010, Egeth et al., 1984, McLeod et al., 1991). Finlayson et al. (2013) suggested that, in the absence of preattentive segmentation, segmenting items according to their depth might be achieved using the same attentional resources required to serially attend to items in a display. If this is the case, the same cortical regions could be recruited in both the target-known and target-unknown conditions, masking their involvement in segmenting the items according to depth.

In terms of top-down activation, driving attentional selectivity to part of the segmented display, we found that the posterior cingulate was engaged when target location was known (*p* < 0.001), as has been found previously (Dent et al., 2011). We also used an ROI analysis to look for segmentation-related activation in the precuneus region which is commonly activated by temporal (preview) and motion segmentation (Dent et al., 2011). However, we found that this specific region was not sensitive to knowledge of target location. The role of the precuneus may be sensitive to the specific task being performed. Dent et al. (2011) compared activation when displays were segmented by motion (moving/stationary items) or time (preview/search displays), with activation when displays were unsegmented (stationary items that appeared simultaneously). In contrast, here we presented segmented displays (items in front and behind fixation) and manipulated knowledge of target location. Both studies found a similar behavioural advantage from being able to attend to a subset of items, but the precuneus may have been specifically involved in segmenting the display rather than guiding attention within an already-segmented display.

We may have found a different pattern of results if the attended depth changed from trial to trial, rather than being consistent within a block of trials. It is likely that trial-to-trial changes would have led to increased activation in the fronto-parietal attention network (Corbetta and Shulman, 2002). It is less clear how this would have affected activation in visual regions: directing attention to one location would have increased activation in regions responsive to that location (Kanwisher and Wojciulik, 2000), but this may have been partially offset by adaptation to repeated stimuli at that location (Grill-Spector et al., 1999).

## Conclusion

Our findings demonstrate that dividing a 3D search display into relevant and irrelevant items is predominantly achieved through activation in depth-sensitive regions along the intraparietal sulcus, rather than filtering in depth-sensitive visual regions. The results also indicate that segmenting the items in depth, in this task at least, is cue invariant: behavioural and neuroimaging findings showed little difference when depths were defined by disparities or surfaces, or when items fell within depth-defined 3D regions.

Supplementary data to this article can be found online at http://dx.doi.org/10.1016/j.neuroimage.2015.07.052.

## Figures and Tables

**Table 1:** Results of the individual analyses of variance (ANOVAs) for each of the depth-selective regions of interest (ROIs).

ROI	display type vertical vs tilted	letter placement planes vs jittered	target knowledge depth known vs unknown
KO	2.46	0.69	0.24
LO	6.21*	1.09	2.06
MT/MT +	2.87	0.00	0.00
VIPS/V7	0.31	0.07	1.54
POIPS	0.14	0.06	3.86
DIPSM	0.01	1.93	5.28*
DIPSA	0.85	0.76	15.31**
phAIP	0.12	0.56	11.33**
Precuneus	0.00	1.07	0.00

Values indicate *F* values with 1 and 15 degrees of freedom. *p < 0.05, **p < 0.0056. ANOVAs probed display type × letter placement × target-knowledge condition, but only the main effects are shown as there were no significant interactions. KO, kinetic occipital area; LO, lateral occipital cortex; MT/MT +, motion area; VIPS, ventral intraparietal sulcus (IPS); POIPS, posterior-occipital IPS; DIPSM, dorsal medial IPS; DIPSA, dorsal anterior IPS. phAIP: putative human anterior IPS.

**Table 2:** Results of the whole-brain analysis. *p* < 0.001, cluster size > 10 voxels.

Region	MNI coordinates	z score	cluster size (voxels)
Target location known > unknown			
Left inferior parietal lobe	− 43, − 54, 55	3.96	51
Right inferior parietal lobe	47, − 50, 52	3.62	19
Posterior cingulate	− 10, − 20, 32	4.46	31
Right cerebellum	22, − 72, − 35	3.70	38
Target location unknown > known			
No significant regions			
Vertical displays > tilted displays			
Posterior visual cortex	7, − 67, 2	3.76	12
Tilted displays > vertical displays			
Posterior visual cortex	− 16, − 84, − 5	5.57	764
Letters in planes > jittered			
No significant regions			
Letters jittered > in planes			
Left supramarginal gyrus	− 36, − 52, 28	3.72	15
Right supramarginal gyrus	27, − 40, 38	3.44	10

## Footnotes

1 We are grateful to Andrew Welchman and Zoe Kourtzi for allowing us to use their localiser tasks.
